# Identification of ultraviolet B radiation-induced microRNAs in normal human dermal papilla cells

**DOI:** 10.3892/mmr.2014.2418

**Published:** 2014-07-24

**Authors:** HWA JUN CHA, OK-YEON KIM, GANG TAI LEE, KWANG SIK LEE, JAE HO LEE, IN-CHUL PARK, SU-JAE LEE, YU RI KIM, KYU JOONG AHN, IN-SOOK AN, SUNGKWAN AN, SEUNGHEE BAE

**Affiliations:** 1 Korea Institute for Skin and Clinical Sciences, Konkuk University, Seoul 143-701, Republic of Korea; 2 Molecular-Targeted Drug Research Center, Konkuk University, Seoul 143-701, Republic of Korea; 3 Coreana Cosmetics Co., Ltd., Cheonan-si, Chungcheongnam-do 330-833, Republic of Korea; 4 Laboratory of Molecular Oncology, Cheil General Hospital and Women’s Healthcare Center, Kwandong University, College of Medicine, Seoul 100-380, Republic of Korea; 5 Laboratory of Functional Genomics, Korea Institute of Radiological and Medical Sciences, Seoul 139-706, Republic of Korea; 6 Department of Chemistry, Hanyang University, Seoul 133-791, Republic of Korea; 7 Department of Dermatology, Konkuk University School of Medicine, Seoul 143-701, Republic of Korea

**Keywords:** microRNA, ultraviolet B, human dermal papilla cells, microarray

## Abstract

Ultraviolet (UV) radiation impairs intracellular functions by directly damaging DNA and by indirectly generating reactive oxygen species (ROS), which induce cell cycle arrest and apoptosis. UV radiation can also alter gene expression profiles, including those of mRNA and microRNA (miRNA). The effects of UV radiation on cellular functions and gene expression have been widely documented in human skin cells such as keratinocytes, melanocytes and dermal fibroblasts, but the effect it has on other types of skin cell such as dermal papilla cells, which are crucial in the induction of hair follicle growth, remains unknown. In the current study, the effect of UV radiation on physiological changes and miRNA-based expression profiles in normal human dermal papilla cells (nHDPs) was investigated. UVB radiation of ≥50 mJ/cm^2^ displayed high cytotoxicity and apoptosis in a dose-dependent manner. In addition, ROS generation was exhibited in UVB-irradiated nHDPs. Furthermore, using miRNA microarray analysis, it was demonstrated that the expression profiles of 42 miRNAs in UVB-irradiated nHDPs were significantly altered compared with those in the controls (35 upregulated and 7 downregulated). The biological functions of the differentially expressed miRNAs were studied with gene ontology analysis to identify their putative target mRNAs, and were demonstrated to be involved in cell survival- and death-related functions. Overall, the results of the present study provide evidence that miRNA-based cellular mechanisms may be involved in the UVB-induced cellular response in nHDPs.

## Introduction

Previous studies have established that chronic exposure to solar radiation leads to skin damage (1,2). Ultraviolet (UV) radiation, which can be categorized into UVA, UVB and UVC according to wavelength, has the potential to cause DNA damage, leading to sunburn and skin cancer (3). UVC radiation has the shortest wavelength and thus emits the highest energy levels (4), but the majority of UVC from sunlight is absorbed by the atmosphere, in particular the ozone layer, so is not a threat to health. UVA and UVB, however, reach the skin by penetrating the atmosphere (5), and in hair follicles, androgenetic alopecia is a result of UV-induced photo-aggravated dermatitis (6). Additionally, UV represses growth and cycling of hair follicles and follicular melanogenesis *in vitro* (7).

microRNAs (miRNAs) are small, non-coding RNAs that regulate mRNA translation (8,9) and have been implicated in the regulation of apoptosis, survival and differentiation (9). UV radiation has been demonstrated to regulate miRNAs in various types of cell (10,11), including melanocytes, in which UV-induced miR-145, miR-148 and miR-25 regulate pigmentation by repressing Myo5a and MITF (12,13). miR-125b and miR-22 promote cell survival by targeting p38α and PTEN following UV irradiation (10,14). Additionally, Pothof *et al* (11) implicated miRNA-mediated gene interference in the UV-induced DNA damage response. In other studies, miRNA expression in UV-irradiated mouse epidermis and human keratinocytes was profiled via microarray analysis (15,16). However, despite these studies, changes in miRNA expression in response to UV radiation remain unclear in human dermal papilla cells. Therefore, in the present study, global miRNA expression in UVB-irradiated human dermal papilla cells was profiled and bioinformatics were utilized to identify putative miRNA target genes and their associated biological functions. The data from the current study may provide insights into a novel mechanism of UV-dependent damage in human dermal papilla cells.

## Materials and methods

### Cell culture and UVB irradiation

Normal human dermal papilla cells (nHDPs) were obtained from Cell Engineering For Origin (Seoul, Korea). nHDPs were maintained in Dulbecco’s modified Eagle’s medium (DMEM; Gibco, Invitrogen Life Technologies, Carlsbad, CA, USA) supplemented with 10% fetal bovine serum (FBS; Sigma-Aldrich, St. Louis, MO, USA), 5,000 U/ml penicillin G and 5,000 μg/ml streptomycin. The cells were incubated at 37˚C in 5% CO_2_ humidity.

Cells were exposed to UVB radiation using a G8T5E lamp (Sankyo Denki, Toshima, Japan). Doses were measured with a UV light meter (Lutron UV-340; Lutron Electronic Enterprise Co., Ltd., Taipei, Taiwan). nHDPs were seeded in 60-mm culture dishes and incubated for 24 h, then washed and resuspended in phosphate-buffered saline (PBS) prior to exposure to UVB. The cells were placed in fresh medium following irradiation. Non-exposed control samples were maintained in the dark under the same conditions.

### RNA extraction and miRNA microarray

All materials were obtained from Agilent Technologies (Santa Clara, CA, USA) unless otherwise stated. Total RNA was extracted using RiboEx™ (Geneall, Seoul, Korea) according to the manufacturer’s instructions. RNA stability was confirmed using the Bioanalyzer 2100. Nucleic acid purity was calculated from A260/A280 and A260/A230 ratios using the MaestroNano spectrophotometer (Maestrogen, Las Vegas, NV, USA). miRNA expression profiles were analyzed using the SurePrint G3 Human v16.0 miRNA 8x60K Microarray kit (based on miRBase release 19.0), which included 1,368 probes representing 1,205 human miRNAs. Total RNA (100 ng) was dephosphorylated using calf intestine alkaline phosphatase (CIP) and denatured by heat inactivation with dimethyl sulfoxide (DMSO). The dephosphorylated RNA was labeled with pCp-Cy3 using T4 RNA ligase. Unlabeled pCp-Cy3 was removed using the Micro Bio-Spin P-6 column (Bio-Rad, Hercules, CA, USA). Labeled RNA was dried and resuspended in Hi-RPM hybridization buffer prior to hybridization with the microarray at 55˚C and 20 rpm for 20 h in an Agilent Microarray Hybridization Oven (Agilent Technologies, Santa Clara, CA, USA). Following hybridization, microarray slides were washed with wash buffers 1 and 2 and then scanned using an Agilent SureScan Microarray Scanner (Agilent Technologies). The scanned image was quantitated using Agilent Feature Extraction Software (version 10.7; Agilent Technologies). The data were analyzed using GeneSpring GX version 11.5 software.

### miRNA target gene prediction and gene ontology (GO) analysis

The target genes of miRNAs that exhibited altered expression levels in response to UVB exposure were predicted using TargetScan (http://www.targetscan.org). The target genes were predicted from the high context score percentile (50–100) in the conserved and nonconserved database. The predicted target genes underwent GO analysis to identify their associated biological functions.

### Cell viability

nHDPs were seeded into a 96-well plate at a density of 5x10^3^ cells/well and incubated for 24 h. The cells were irradiated with different doses of UV (0–400 mJ/cm^2^) and incubated for another 24 h. The cells were then incubated with 0.5 mg/ml 3-(4,5-dimethylthiazol-2-yl)-2,5-diphenyltetrazolium bromide (MTT) dissolved in DMSO for 1 h. The absorbance at 490 nm was measured with an iMark Microplate Absorbance reader (Bio-Rad).

### Cell cycle analysis

nHDPs were plated on 60-mm tissue culture dishes at a density of 2x10^6^ cells/plate and then grown until 70% confluent, prior to irradiation with different doses of UVB (0–400 mJ/cm^2^). Following the 24-h incubation, the cells were trypsinized and fixed with 70% ethanol for 18 h at 4˚C. The cells were then resuspended in 1 ml propidium iodide (PI) staining solution (50 μg/ml PI, 0.1 μg/ml RNase, and 0.05% Triton X-100 in PBS) for 1 h. The stained cells were analyzed using a FACSCalibur flow cytometer (BD Biosciences, San Jose, CA, USA). A minimum of 10,000 events were collected in each analysis. The various cell cycle populations were determined by ModFit LT (Verity Software House, Topsham, ME, USA).

### Reactive oxygen species (ROS) staining

Intercellular ROS levels were measured using 2′,7′-dichlorofluorescein diacetate (DCF-DA) (Sigma-Aldrich) as previously described (17). nHDPs were plated onto 60-mm tissue culture dishes at a density of 2x10^6^ cells/plate. The cells were irradiated with UVB and then incubated for 24 h prior to staining with 20 μM DCF-DA for 30 min. The stained cells were analyzed via flow cytometry using the FACSCalibur flow cytometer.

### Statistical analysis

Statistical significance was determined by the Student’s t-test and data were subjected to global normalization. P<0.05 was considered to indicate a statistically significant difference.

## Results

### UVB irradiation decreases cell viability by increasing the occurrence of cell cycle arrest or apoptosis in nHDPs

**Figure 1:**
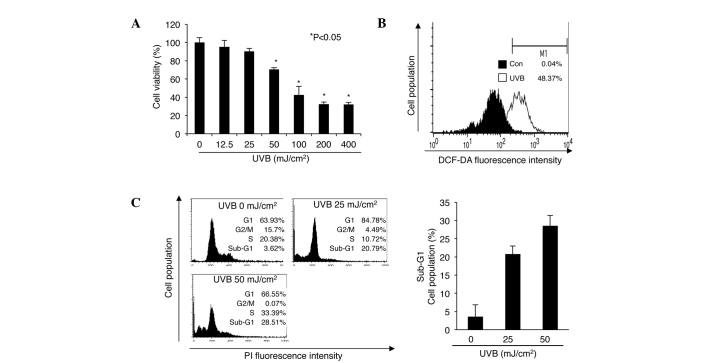
Effect of UVB irradiation on cell viability, ROS production and the cell cycle in nHDPs. (A) Effect of UVB radiation on nHDP viability. Cell viability was measured by MTT assay. Results are presented as the mean ± standard error of the percentage of control OD of triplicate samples. ^*^P<0.05 vs. 0 mJ/cm^2^ UVB irradiation. (B) Effect of UVB on the levels of ROS in nHDPs. Different cell populations are represented by different colors (black, 0 mJ/cm^2^ UVB; white, 50 mJ/cm^2^ UVB). (C) Effect of UVB on the cell cycle in nHDPs. The distributions of cells of different populations in the different stages of the cell cycle were analyzed by flow cytometry using PI-stained nHDPs. The frequency of cells in the sub-G1 phase is presented as the mean ± standard error of the percentage of the gated cell population of triplicate samples. UVB, ultraviolet B; DCF-DA, 2′,7′-dichloroflorescein diacetate; PI, propidium iodide; ROS, reactive oxygen species; nHDP, normal human dermal papilla cell; OD, optical density.

Agents such as UV radiation, that induce DNA damage in mammalian cells, trigger growth arrest, cell cycle arrest, and apoptosis by regulating the ATM-p53 pathway (18,19). However, different types of cell exhibit varied responses to equivalent UV doses (5,20–23). Therefore, in the current study, to determine how nHDPs respond to UV irradiation, these cells were exposed to 0–400 mJ/cm^2^ of UVB radiation for 24 h, prior to a cell viability assessment (Fig. 1A). Cell viability was significantly reduced to 70.34% following exposure to 50 mJ/cm^2^ of UVB compared with that following exposure to 0 mJ/cm^2^. As previous studies have shown that UVB radiation regulates cell viability and apoptosis by increasing ROS production (24,25), intracellular ROS was examined using DCF-DA in the current study. The results demonstrated that ROS production increased in cells exposed to 50 mJ/cm^2^ UVB compared with those exposed to 0 mJ/cm^2^ (Fig. 1B). Analysis of cell cycle progression in cells that were exposed to 0, 25 and 50 mJ/cm^2^ UVB for 24 h revealed that G1-phase cell cycle arrest was induced by 25 mJ/cm^2^ UVB irradiation (Fig. 1C). Notably, the frequency of sub-G1 cells, which represent apoptotic cells, was increased in nHDPs exposed to 50 mJ/cm^2^ UVB compared with those exposed to 0 mJ/cm^2^.

### miRNA expression is altered by UVB irradiation

**Figure 2:**
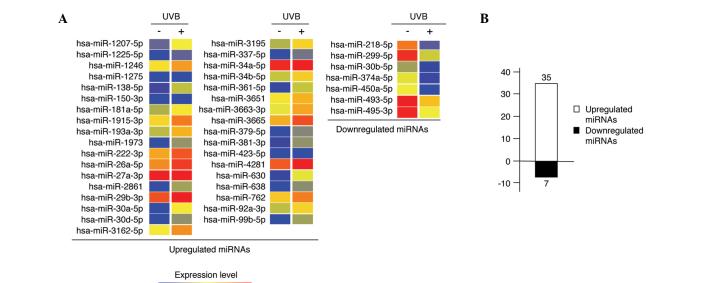
UVB radiation alters miRNA expression profiles in nHDPs. (A) Heat-map of miRNAs whose expressions levels were up- and downregulated >1.5-fold in 50 mJ/cm^2^ UVB-irradiated nHDPs vs. non-irradiated nHDPs. The color bar displaying fluorescence intensity corresponds to miRNA expression levels. Highly expressed miRNAs are red, while those present at low levels are blue. (B) Total number of miRNAs up- and downregulated by 50 mJ/cm^2^ UVB radiation in nHDPs. UVB, ultraviolet B; miRNA, microRNA; nHDP, normal human dermal papilla cell.

To analyze UVB-dependent changes in miRNA expression in nHDPs, miRNA microarray analysis was performed using arrays containing 1,368 probes representing 1,205 human miRNAs. Total RNA was extracted from non-irradiated and 50 mJ/cm^2^ UVB-irradiated nHDPs. Data from each sample were subjected to global normalization. To obtain refined data, miRNAs were selected and further considered as a result of flag-present filtration in the data file, indicating that the sensitivity of the selected miRNAs were sufficient for the microarray. A total of 183 miRNAs were selected for further analysis. miRNAs from 50 mJ/cm^2^ UVB-irradiated nHDPs that were upregulated (n=35) and downregulated (n=7) at least 1.5-fold compared with those of the non-irradiated control nHDPs were identified (Fig. 2), and lists of these 42 miRNAs are presented in Tables I and II.

### GO analysis of UVB-specific putative miRNA target genes in nHDPs

**Figure 3:**
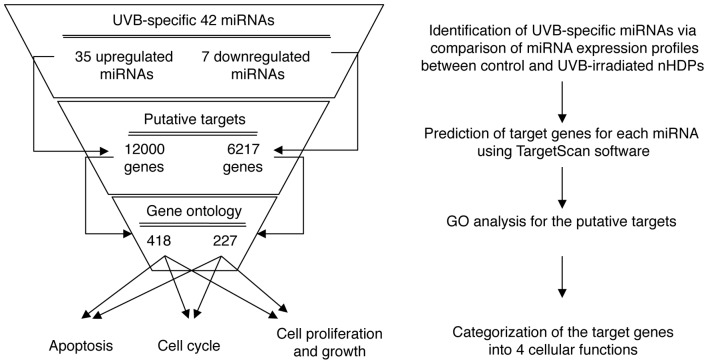
Scheme of bioinformatic analysis performed. miRNAs whose expression profiles were altered by UVB irradiation were selected by comparing the microarray data of non-irradiated and 50 mJ/cm^2^ UVB-exposed nHDPs. Predicted targets of the UVB-specific miRNAs were determined using TargetScan software. The target genes were then categorized into three groups, namely apoptosis, cell cycle, and cell growth and proliferation by performing GO analysis. UVB, ultraviolet B; miRNA, microRNA; nHDP, normal human dermal papilla cell; GO, gene ontology.

To identify the cellular functions associated with UVB-mediated changes in the levels of miRNA expression, a multi-step bioinformatics scheme was used to predict putative miRNA target genes and their biological functions (Fig. 3). TargetScan, a sequence-based miRNA target prediction database, was used to identify 18,217 putative target genes for the up- and downregulated miRNAs. The analysis revealed 12,000 potential targets for the 35 upregulated miRNAs and 6,217 targets for the 7 downregulated miRNAs. Subsequently, the putative target genes involved in UV-mediated cellular damage, including the cell cycle, apoptosis, cell growth and proliferation, were screened. Using a GO web-based tool, AmiGO, the gene lists for the above four GO terms (cell cycle, apoptosis, cell growth and proliferation) were obtained, and these genes were compared with the putative target genes. The overlapping genes in the two groups were then identified (Fig. 3). Among the deregulated 42 miRNAs (Table I and II), the majority of miRNAs were altered >2.0-fold. Thus, the five most upregulated and downregulated miRNAs were selected to obtain more representative biological meanings for the putative genes. The predicted target genes of the top 5 most differentially upregulated and downregulated miRNAs in the UVB-irradiated HDPs are categorized by their functions in Tables III and IV, respectively.

**Figure 4:**
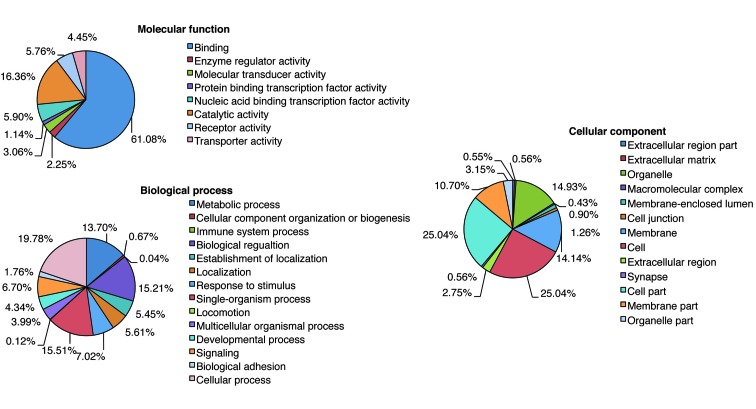
GO analysis of predicted target genes. miRNA target genes were predicted using web-based TargetScan software. Biological roles of each target were analyzed by GO. A diagrammatic representation of the GO analysis was produced using GeneSpring GX. GO, gene ontology; miRNA, microRNA.

**Figure 5:**
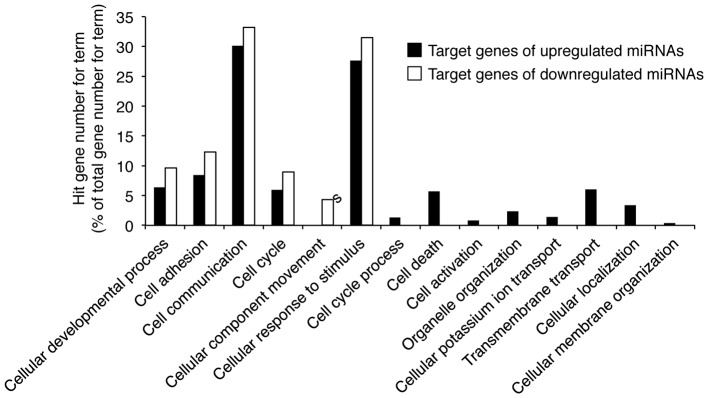
Detailed GO analysis of cellular process, which was the highest regulated subsection of the biological process category.

Each putative target gene was subjected to GO analysis to reveal its cellular function. GO was analyzed using three significant categories as follows: Molecular function, biological process and cellular component. The analysis revealed a wide distribution of cellular functions, which are presented in Fig. 4. Cellular process, which comprised the largest percentage of the biological process category in Fig. 4, was analyzed in greater detail. The highest percentage of gene functions were in the cellular process category, which encompasses the processes analyzed in Fig. 5. In particular, the target genes of miRNAs up- and downregulated by UVB radiation are associated with cellular responses to stimuli and cell communication. These results suggest that UVB-induced growth arrest and apoptosis may be mediated by miRNAs involved in cellular stimulation and communication in nHDPs.

## Discussion

Sunlight is a multi-wavelength light that modulates cellular signaling in the skin (3). UV radiation from sunlight is known to induce DNA damage in types of skin cell such as keratinocytes and fibroblasts (26), but the UV-mediated cellular effects in nHDPs have not yet been reported. In the current study it was determined that UVB radiation represses nHDP growth via cell cycle arrest and apoptosis, and that it can induce ROS generation. miRNAs have been demonstrated to have a major influence over the control of UVB-induced growth repression in a previous study (27). Therefore, in the present study, the miRNA expression profile in nHDPs following UVB-irradiation was analyzed. A total of 42 miRNAs whose expression in nHDPs changed at least 1.5-fold following UVB irradiation were identified. One of the upregulated miRNAs identified in the current study was miR-30a-5p, which has been reported to modulate cell growth by targeting the denticleless protein homolog, a gene implicated in S phase and UVB-induced growth arrest (28). miR-34a-5p and miR-34b-5p, which were upregulated 1.6- and 1.8-fold in the current study, respectively, are induced by DNA damage in various types of cell (29,30), and by p53, which is activated by DNA break-induced ataxia telangiectasia mutated activation (29). Overall, the results of the current study indicate that, in nHDPs, UVB regulates specific miRNAs in order to regulate cell growth and death.

In addition, the present study identified putative target genes of the up- and downregulated miRNAs, and categorized their reported biological functions by GO into cell cycle, apoptosis and cell growth and proliferation categories. UV irradiation of various types of cell, including keratinocytes, melanocytes and dermal fibroblasts, can regulate cell fate via the intrinsic apoptosis pathway (26,31,32). Consistent with this, the results of the present study demonstrated that the miRNAs that were upregulated by UVB irradiation targeted intrinsic apoptosis pathway-related genes, including NAIP (targeted by miR-30a-5p), XAF1 (targeted by miR-638) and XIAP (targeted by miR-630). In addition, miRNAs induced by UVB exposure were demonstrated to regulate core cell cycle regulators including cyclins, cyclin dependent kinases (CDKs) and CDK inhibitors. For example, upregulated miRNAs targeted CDC7 (targeted by miR-30a-5p and miR-630) and CDK2 (targeted by miR-638), while downregulated miRNAs targeted CDKN1A (targeted by miR-299-5p), CDKN1B (targeted by miR-218-5p and miR-495-3p) and CCNA2 (targeted by miR-218-5p).

Overall, data of the current study demonstrated that miRNA expression in nHDPs was altered in response to UVB exposure. This furthers the current understanding of the cellular mechanisms mediating the response to UVB exposure, whilst providing insight into the processes involved in sunlight-induced hair and skin aging.

## Figures and Tables

**Table I:** miRNAs upregulated at least 1.5-fold in UVB-irradiated nHDPs.

miRNA	Fold change	Chr
hsa-miR-1207-5p	2.00	chr8
hsa-miR-1225-5p	1.62	chr16
hsa-miR-1246	1.79	chr2
hsa-miR-1275	1.76	chr6
hsa-miR-138-5p	1.80	chr3
hsa-miR-150-3p	2.24	chr19
hsa-miR-181a-5p	1.56	chr1
hsa-miR-1915-3p	2.19	chr10
hsa-miR-193a-3p	2.32	chr17
hsa-miR-1973	2.18	chr4
hsa-miR-222-3p	2.07	chrX
hsa-miR-26a-5p	2.08	chr3
hsa-miR-27a-3p	1.90	chr19
hsa-miR-2861	2.42	chr9
hsa-miR-29b-3p	2.25	chr1
hsa-miR-30a-5p	4.03	chr6
hsa-miR-30d-5p	2.28	chr8
hsa-miR-3162-5p	2.20	chr11
hsa-miR-3195	1.85	chr20
hsa-miR-337-5p	1.62	chr14
hsa-miR-34a-5p	1.64	chr1
hsa-miR-34b-5p	1.76	chr11
hsa-miR-361-5p	2.12	chrX
hsa-miR-3651	1.55	chr9
hsa-miR-3663-3p	2.16	chr10
hsa-miR-3665	2.34	chr13
hsa-miR-379-5p	2.09	chr14
hsa-miR-381-3p	1.51	chr14
hsa-miR-423-5p	1.78	chr17
hsa-miR-4281	2.09	chr5
hsa-miR-630	2.35	chr15
hsa-miR-638	2.55	chr19
hsa-miR-762	1.89	chr16
hsa-miR-92a-3p	1.74	chr13
hsa-miR-99b-5p	3.28	chr19

miRNA, microRNA; UVB, ultraviolet B; nHDP, normal human dermal papilla cell; Chr, chromosome.

**Table II:** miRNAs downregulated at least 1.5-fold in UVB-irradiated nHDPs.

miRNA	Fold change	Chr
hsa-miR-1207-5p	2.00	chr8
hsa-miR-1225-5p	1.62	chr16
hsa-miR-1246	1.79	chr2
hsa-miR-1275	1.76	chr6
hsa-miR-138-5p	1.80	chr3
hsa-miR-150-3p	2.24	chr19
hsa-miR-181a-5p	1.56	chr1

miRNA, microRNA; UVB, ultraviolet B; nHDP, normal human dermal papilla cell; Chr, chromosome.

**Table III:** Predicted target genes of the top five most differentially upregulated miRNAs in UVB-irradiated nHDPs.

	Target genes and functions		
	
miRNA	Cell cycle	Apoptosis	Cell growth and proliferation
hsa-miR-30a-5p	APBB2, RHOB, EPHB2, NF1 NOTCH2, PNN, TSC1, RECK, CUL3, CUL2, BCL10, MTBP, TBRG1, TBRG1	ACTC1, CASP3, GJA1, HTT, IL1A, IL2RA, IL17A, MAP3K5, MLL, NAIP, PTGER3, ATXN1, TFDP1, TIA1, UNC5C, TNFSF9, TNFRSF10D, BCL10, ATG12, EBAG9, ARHGEF6, ATG5, BCL2L11, EDAR, TRIM35, SIRT1, TCTN3, CECR2, SH3KBP1, DDIT4, C8orf4, AVEN, BIRC6, TRIB3, NLRP3, RFFL, TICAM1, PRUNE2, RNF144B, BCL2L15	ADRA1D, ADRA2A, ADRB2, IL7, TLX1, BNC1, DDX11, CAMK2D, JAG2, KRAS, LIFR, LYN, MAFG, PDGFRB, PPP2CA, SOX9, VIPR1, CDC7, CUL3, SOCS1, CREG1, NOV, SOCS3, EBAG9, CFDP1, ENOX2, C19orf10, BIRC6, CDCA7
hsa-miR-99b-5p	MCC, RASSF4	DFFB, NLRP2, RNF144B	BMPR2, IGF1R, STAT5B
hsa-miR-638	CDKN2B, MCC, MPHOSPH1, XAF1	CLU, ATN1, TNFRSF11B, PAK2, TNFRSF1B, ATG5, CIDEB, SAP30BP, XAF1, UBE2Z, FAM130A1, RHOT2, RFFL	CD47, CDK2, CLU, CTF1, GAP43, IFNG, IL11, LIF, LIFR, PAK2, TRAF5, VEGFA, HOXB13, BRD8
hsa-miR-2861	-	-	-
hsa-miR-630	RHOB, CDKN2B, CYLD, NOTCH2, RB1CC1, WWOX, ZAK, C11orf82, LIN9	XIAP, DOCK1, EP300, GJA1, PAX3, TP63, ATG12, SLK, SMNDC1, NCKAP1, CKAP2, CROP, ZAK, DDIT4, AVEN, ZMAT3, TP53INP1, C11orf82	BMPR2, KLF5, FOXO1, IGF1R, CYR61, IL7, SSR1, TDGF1, TGFBR2, CDC7, SOCS2, RBM9, CCDC88A, ZMAT3

miRNA, microRNA; UVB, ultraviolet B; nHDP, normal human dermal papilla cell.

**Table IV:** Predicted target genes of the top five most differentially downregulated miRNAs in UVB-irradiated nHDPs.

	Target genes and functions		
	
miRNA	Cell cycle	Apoptosis	Cell growth and proliferation
hsa-miR-218-5p	TRIM13, CDK6, CDKN1B, KHDRBS1, CETN2, MAPRE2, RCC1, FOXN3, PYHIN1, DCC, E2F2, SENP5, FANCD2, MAPRE3, SEPT2, SASH1, CLASP1, SPECC1L, SH3BP4, EGFL6, PDCD4, HLA, QB1, HPGD, BIRC5, MCC, NEDD9, ZAK, ANLN, PPP1CB, PPP1CC, RIF1, RCBTB1, FANCI, SMPD3, PCNP, CCND1, SIAH2, BRCA1, TACC1, PTP4A1, MAP9, RASSF5, PARD6B, CCNA2, CCND3, LYK5, RASSF2	-	NAMPT, CDKN1B, NET1, SOCS4, CTGF, FLT1, KIT, KRT6A, LIF, LIFR, MST1R, NEDD9, NODAL, NRAS, PAK2, C20orf20, BIRC6, PURA, APPA2, BNC1, SHC1, SSR1, TRAF5, YEATS4, CUL3, SOCS3, SOCS6, HTRA3, CD47, SOCS5
hsa-miR-450a-5p	RCC1, EGFR, CD2AP, XAF1	-	-
hsa-miR-299-5p	CDKN1A, MAEA, SMC2, CTCF, SPIN1, POLS, CEP110, FOXN3, GADD45A, AHR, SENP5 , FANCD2, SIRT2, SEP2, CCNDBP1, CD2AP, RABGAP1, KIF11, NF1, NPAT, ZAK, MPHOSPH8, PPP1CB, CEP55, MTUS1, RAD21, RAP1A, AVPI1, SIAH1, PTP4A1, CDC7, PPAPDC1B, MCM8, TBRG1, CDC16, CCNG1, CCNG2, CCNH, AURKB, HDAC4	-	IGFBP3, IL8RB, NOV, SIRPG, SSR1, TDGF1, CDC7, SOCS6, CD86, SOCS5
hsa-miR-374a-5p	CDK6, SPIN1, POLS, ZWINT, ADCYAP1, ESCO2, SLC5A8, GADD45A, AHR, CCRK, CD2AP, NIPBL, EGFL6, APPL1, GAS1, MTBP, LIN9, ANXA1, HELLS, HPGD, FLJ44060, ING1, LOH11CR2A, MCM2, MCM6, MLH1, NBN, ATM, NEDD9, NEK2, NF1, ZAK, ANLN, ERBB2IP, MAPK1, MAPK6, MAPK7, PCNP, PTEN, SPC25, ARHGAP20, CCND1, NCAPG, PAPD5, BMP2, NEK4, TACC1, TFDP1, TP53BP2, SUV39H2, MAP9, CHAF1B, RECK, MCM8, PARD6B, TBRG1, CDC14A, BCL10, STARD13, CCNE2, KNTC1	-	NAMPT, DNAJA2, UBE2E3, CHAD, CLU, SOCS4, HES1, IGFBP3, IGFBP7, IL7, CXCL10, KRAS, LIFR, MYC, NEDD9, PAK2, CRIM1, PURA, BCL2, PAPPA2, CXCL5, SLAMF1, BTC, TBX3, TSHR, VEGFA, YEATS4, SOCS6, CIAO1, NTN1, CD47, SOCS5
hsa-miR-495-3p	CDKN1A, MAEA, SMC2, CTCF, SPIN1, POLS, CEP110, FOXN3, GADD45A, AHR, SENP5, FANCD2, SIRT2, SEP2, CCNDBP1, CD2AP, RABGAP1, KIF11, NF1, NPAT, ZAK, MPHOSPH8, PPP1CB, CEP55, MTUS1, RAD21, RAP1A, AVPI1, SIAH1, CD2AP, SH3BP4, CADM1, ANAPC13, GAS1, LIN9, UHRF1, HHEX, TMPRSS11A, ING1, JAG2, LIG4, AD2L1, MCC, MCM2, MCM3, MCM7, NBN, EDD9, PNN, ANLN, XRN1, TXNL4B, TIPIN, PPP1CB, RIF1, RCBTB1, PPP6C, CCAR1, SEP11, BIN3, ERBB2IP, KLK10, PCNP, PTCH1, PTEN, SPC25, ARHGAP20, RAD17, RAP1A, CCND1, NCAPG, PAPD5, SIAH1, BMP2, NEK4, TACC1, BUB1, TFDP1, WEE1, EVI5, HMGA2, CDC7, RASSF5, CUL4B, PARD6G, JUB, CDC14A, RUNX3, PCAF, BCL10, UBA3, CCNT2, PKMYT1, CCNE2, ZNF830, CCPG1, WTAP, MDC1, HDAC4, DLGAP5, MTSS1, RB1CC1, CDC2, TLK1, CDC6, DLEC1, CASP8AP2, SEP7	-	CDKN1B, NET1, TNFSF13B, MORF4L1, ESM1, FGFR1OP, SOCS4, ADM, ADRB2, DDX11, S1PR3, FGF7, TMEM97, ID4, IFNG, IGF1, IGFBP3, IL6, IL8RB, IL11, LIFR, NAP1L1, NDN, NEDD9, NRAS, CRIM1, FGFRL1, TIPIN, C19orf10, HTRA1, CXCL5, SSR1, KLF5, TGFB2, TRAF5, VEGFA, HMGA2, CAMK2D, CDC7, BRMS1L, CREG1, FGF17, NRP1, SOCS3, SOCS6, CD47, MORF4L2

miRNA, microRNA; UVB, ultraviolet B; nHDP, normal human dermal papilla cell.
